# Rectangular partition for n-dimensional images with arbitrarily shaped rectilinear objects

**DOI:** 10.1016/j.heliyon.2024.e35956

**Published:** 2024-08-08

**Authors:** Ville Pitkäkangas

**Affiliations:** Centria University of Applied Sciences, Vierimaantie 7, 84100, Ylivieska, Finland

**Keywords:** Computational geometry, Computer vision, Image analysis, Image processing, Polygon partition problem

## Abstract

Partitioning two- or multidimensional polygons into rectangular and rectilinear components is a fundamental problem in computational geometry. Rectangular and rectilinear decomposition have multiple applications in various fields of arts as well as sciences, especially when dissecting information into smaller chunks for efficient analysis, manipulation, identification, storage, and retrieval is essential. This article presents three simple yet elegant solutions for splitting geometric shapes (particularly non-diagonal ones) into non-overlapping and rectangular sub-objects. Experimental results suggest that each proposed method can successfully divide n-dimensional rectilinear shapes, including those with holes, into rectangular components containing no background elements. The proposed methods underwent testing on a dataset of 13 binary images, each with 1 … 4 dimensions, and the most extensive image contained 4096 elements. The test session consisted of 5 runs where starting points for decomposition were randomized where applicable. In the worst case, two of the three methods could complete the task in under 40 ms, while this value for the third method was around 11 s. The success rate for all the algorithms was 100 %.

## Highlights

• Three methods can partition rectilinear shapes into rectangular sub-objects.
• Input shapes can have any dimensionality, concaveness, and holes.
• Output objects are nearly always over one unit long in at least one dimension.
• The methods can be applied in robotics, analytics, simulation, design, and more.

## 1 Introduction

Rectangular and rectilinear partition and decomposition are widely used in applications such as VLSI design [1], DNA microsequencing [1], robotics [1], audio propagation modeling [2,3], bitmap compression [1,[4], [5], [6]], geographic information systems [7], route-planning of autonomous vehicles [8], data compression [5,6], object recognition [6], image processing [6], manufacturing [6], data visualization [9], database management [10], and load balancing [10,11]. It can also be used in metrology, dimensionality reduction, component analysis, part of computer vision algorithms [6], or game or building design.

For metrology and building design, rectangular partitioning can be suitable for splitting an object or a part thereof for more accurate measurements. Applications in data compression, dimensionality reduction, and component analysis could involve decomposing a complex shape or object into rectangles or cuboids and saving only their coordinates and dimensions instead of recording the whole geometry. In computer vision, rectangular partition techniques can be used for feature extraction and as part of other algorithms, such as skeletonization or thinning. Höschl and Flusser [6] mention rectangular decomposition as a tool to speed up feature calculation for describing and recognizing 3D shapes. In game design and simulations, rectangular partition or decomposition has potential applications in, for example, level design, artificial intelligence (navigation and pathfinding), or physics modeling. Four-dimensional rectangular partition could be used to study changes in buildings or other environments over time, or it might be utilized in games or simulations featuring interlinked 3D worlds.

Potential applications may even involve multidimensionality-related disciplines, such as quantum physics and string theory, or they can be part of geometry optimization or any data analytics where multiple variables form interconnected dimensions.

In this article, rectangular partitioning means a process where an object is split into non-overlapping sub-objects consisting only of foreground elements and having 2^n^ vertices, 2^n^*n/2 edges, and 2^(n−2)^*(n!/(2!*(n-2)!)) faces, where n (n ∈ ℕ) is the number of dimensions of the image and the exclamation mark denotes factorial. Simplistically, each extracted sub-object is an n-dimensional equivalent of a cuboid. This article proposes three novel algorithms for partitioning exclusively rectilinear objects consisting of straight lines perpendicular or orthogonal to one another. These objects exist within binary images that are processed as arrays where non-zero values represent parts belonging to objects and zeros are the background. In this study, we call the shapes resulting from partition “extracted (rectangular) shapes,” “sub-shapes,” or “sub-objects”.

A partitioned shape **B** extracted from image **A** can be described as a set of mathematical equations (Equations (1), (2), (3), (4)):

(1) $$\begin{array}{c} A\in R^{n_{1}\times n_{2}\times \ldots \times n_{k}} \end{array}$$

(2) $$\begin{array}{c} B=A\left[{\text{start}}_{1}:{\text{end}}_{1},{\text{start}}_{2}:{\text{end}}_{2},\ldots ,{\text{start}}_{k}:{\text{end}}_{k}\right] \end{array}$$

(3) $$\begin{array}{cc} \forall i,j,\ldots ,k & B\left[i,j,\ldots ,k\right]=B\left[0,0,\ldots ,0\right] \end{array}$$

(4) $$\begin{array}{cc} \forall i,j,\ldots ,k & B\left[i,j,\ldots ,k\right]\neq 0 \end{array}$$

in Equations (1), (2), (3), (4), k represents the number of dimensions in the matrices. In Equation (2), “start” and “end” denote the first and last occurrences of a foreground element (uniquely labeled in the final output) of the extracted shape.

It should be noted that rectangular partition is a process that can lead to multiple results even for the same object (Fig. 1); therefore, there can be multiple correct answers for a single problem. Two of the three partition methods presented in this work rely on randomness, which can introduce variety to outcomes, depending on the position where each partition happens to begin. Partition results can, however, be categorized by their properties, such as the sizes and shapes of the extracted sub-objects. This sub-shape characterization, however, is a separate process that must be done as appropriate by application.

**Fig. 1:**
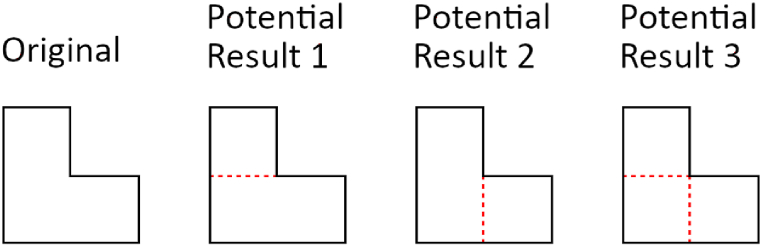
Possible results of rectangular partition: even a simple L-shaped 2D object (first shape from the left) can be decomposed in at least three unique ways (second, third, and fourth shape from the left). Dashed lines denote sub-object edges.

While rectangular partitioning has been studied extensively [[1], [2], [3], [4], [5], [6], [7], [8], [9], [10], [11], [12], [13]], there still are some unexplored areas and unsolved problems in this field. The novelty of the three partition algorithms proposed in this article is their generalizability for n dimensions. Furthermore, they can remarkably decompose arbitrarily shaped rectangular and rectilinear objects, including those with holes and concavities. An additional benefit of these methods is their relative ease of implementation. Therefore, the algorithms presented here aim to provide a solution to a significant problem in computational geometry. All three methods and the related optimization procedure were found independently and are not based on any algorithm existing in the literature.

The article is organized as follows: the introduction is followed by the methods section, where the three solutions, their testing process, and optimization of partition results are presented. After the methods is a separate section that includes reporting the outcomes of the tests. Finally, a section for conclusions ends the article. The expanded outline is below.

1. Introduction
  a. Definition of the problem and its applications
  b. Definitions of key concepts, starting points, and desired outcomes
  c. Challenges and limitations
  d. Introduction of proposed methods and motivation behind them
2. Methods
  2.1. Overview
    a. Introduction of the three proposed methods and objectives
    b. Summary and theory of the methods
    c. Limitations, mitigations, and scope
    d. Implementation details: Programming languages and libraries, key concepts
  2.2. Test Procedure
    a. Test dataset
    b. Test setup
    c. Test process step-by-step
    d. Criteria for a successful and a failed test
  2.3. Solutions
    2.3.1. Special Solution
      a. Introduction, including pros and cons
      b. Brief description
      c. Algorithm
    2.3.2. General Solution I
      a. Introduction, advantages over Special Solution
      b. Main algorithm: overview and steps
      c. Auxiliary algorithms (boundary-finding and cleanup): overview and steps
    2.3.3. General Solution II
      a. Introduction, comparison to General Solution I
      b. Auxiliary algorithms (permutation creation, finding the largest template size, image slicing): overview and steps
      c. Main algorithm: overview and steps
  2.4. Optimization
    a. Conditions for optimization
    b. The optimization process
    c. Cleanup of the results (label reduction, or reassigning unused labels)
    d. Evaluation of the optimization and cleanup results
3. Experimental Results
  a. Evaluation of the success of each proposed method
  b. Benchmarking and statistics, including execution times
  c. Example output images
  d. Interpretation of output images
  e. Brief analysis and discussion
  f. A case for “oblique” objects
4. Conclusions
  a. Summary of the polygon partitioning problem and solutions
  b. Summary of pros and cons of each proposed method
  c. Possible future work

## 2 Methods

### 2.1 Overview

In this article, three solutions for rectangular partition are presented. The first is a solution for cases where the entire image or volume must be iterated. The second and third solutions are more generalizable; they offer random access to the image data and the option to process only part(s) of the image. The proposed methods are named Special, General I, and General II.

The proposed methods use the highest common neighborhood denominator when determining which parts belong in which connected component: lines for 2D, faces for 3D objects, et cetera. The idea is like the concept of hypercubic meshes in Ref. [14]. This type of neighborhood connection is used to ensure there are no holes in the extracted shapes. The output of each solution can be presented as an image of the same shape and size as the input, with a unique label assigned to each extracted rectangular and rectilinear component.

In essence, when using Special and General I methods, the process of rectangular partition consists of three steps: finding the starting point; expanding the selection until background, visited foreground elements, or array edges are reached; and finding holes in the selection and shrinking it until there are no more holes in the region. Without the last step, the extraction result would resemble a convex hull, covering all holes and possibly even dents of the object. The General II method differs from the other two because it relies on template matching.

The proposed techniques have a few limitations. The input must be provided as bitmaps, rather than n-dimensional meshes or vector graphics. The solutions are designed for dimensionally optimized input arrays, meaning all dimensions must have a length greater than one. In addition, while extracted shapes are rectangular, the outcome may not be optimal or fit for a particular purpose. In other words, even though the output only has rectangular objects, the objective of the partitioning process is not to explicitly find or use the smallest, the largest, the most, or the fewest shapes. However, it was still considered beneficial for potential applications that the partitioned shapes have more than one unit of length in all dimensions whenever possible. An optimization algorithm was, therefore, developed to minimize the number of extracted objects. Since the generalizable solutions rely on randomness, the results may vary on each execution. Shapes inside or overlapping bounding boxes of other shapes may impose another limitation due to the possibility of these separate but close objects being mistakenly recognized as parts of each other. This problem can be solved by applying connected-component labeling on the image before attempting to partition it, copying each labeled object to a blank image, and performing the rectangular partition on each new picture, thus processing each object separately.

The coordinate system used is generally that of the array (image), meaning almost no transforms to local coordinates of the shapes or regions are done, thus resulting in the extracted shapes being always axis-aligned and never tilted according to the orientations of the source objects.

All solutions were implemented in the Python programming language [15] with ease of implementation, high precision and reliability, and dependency on as few software libraries as possible in mind. The only software package the solution implementations depend on is [16], which is used for array operations.

**Fig. 2:**
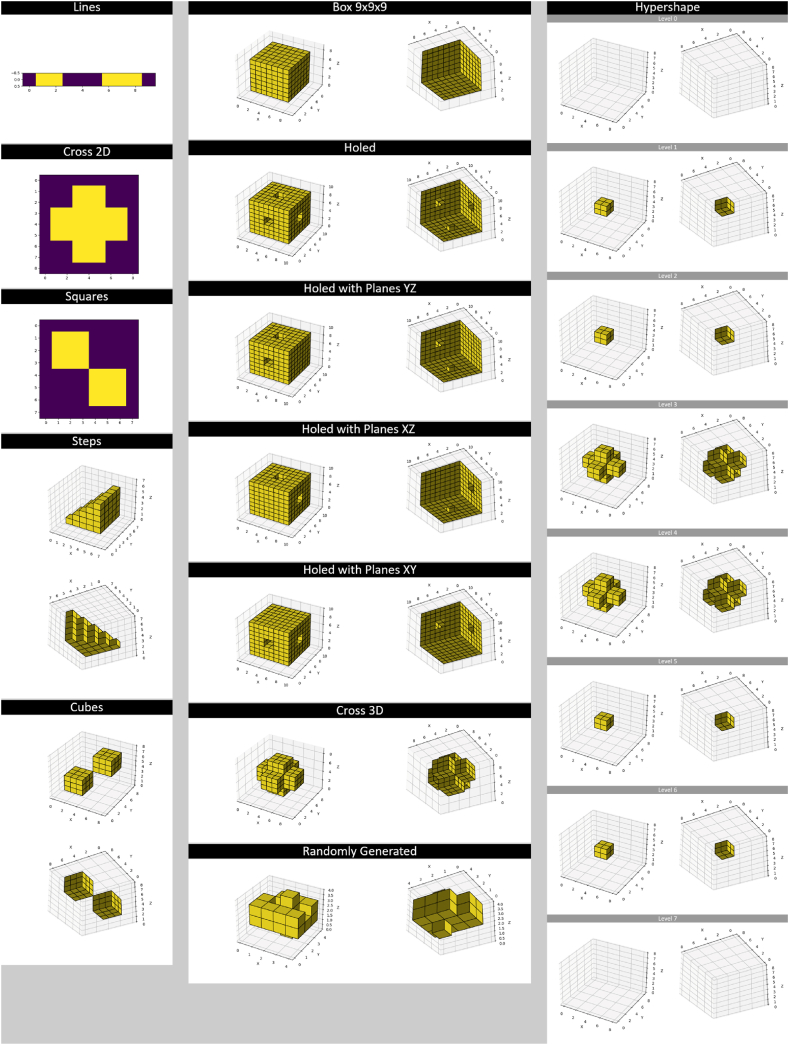
Dataset. Each array has its name written above it. The “Hypershape” array is four-dimensional but split into three-dimensional slices along the w-axis for visualization. “Squares” and “Cross 2D” are two-dimensional, and “Lines” is one-dimensional. All the other arrays are three-dimensional. The purple color in 1D and 2D shapes represents the background.

Matplotlib [17] and Pillow [18] were used for visualizing the datasets and test results for this article. Three-dimensional arrays and slices are shown from two sides; this was realized by saving the view, recording its azimuth and elevation angles, adding 180° to the former and multiplying the latter with −1, redrawing the view using the new angle parameters, and saving the second view. This technique was used for Fig. 2, Fig. 8, Fig. 9.

Algorithms for the proposed methods are presented in Python-like pseudocode in Sections 2.3, 2.4. For readers unfamiliar with the Python language, a glossary of some terms and symbols used in the algorithms is shown in Table 1. In arrays and other iterable variables, indexing starts from zero.

**Table 1:** Glossary of Python terms and symbols used in pseudocode representation of the proposed algorithms.

Keyword(s) or symbol(s)	Definition	Example
[]	empty array (list)	*a = []* Make an empty array named *a*
None	null object or variable	*a = None* Set *a* to null
list	one-dimensional array	*a = list(b)* Convert *b* to a list and assign it to *a* Given *x = [1, 2, 3]*, the type of *x* is a list.
zip	combine iterable variables (such as lists) into a list of tuples	*a = [1, 2, 3]; b = [4, 5, 6]* c = zip(*(a, b)) If *c* was converted to a list and printed, it would show *([(1,4), (2,5), (3,6)])*.
*x	unary star before the variable x is used to unpack variable x; often done when combining variable(s) with zip	*a = [1, 2, 3]; b = [4, 5, 6]* *c = zip((a, b))* Without the * in zip, *c*, when converted to a list, will include *a* and *b* wrapped in tuples: *[([1, 2, 3],), ([4, 5, 6],)]*
np.where	find coordinates that meet the given condition(s)	*coords = np.where(a = = 0)* Find all coordinates of zero elements in *a* and return them to *coords*.
tuple	read-only one-dimensional array; used for example to return “multiple values” from a function and to crop images	*a = tuple(b)* Convert *b* to a tuple and assign it to *a* Given *x = (1, 2, 3)*, the type of *x* is a tuple (notice parentheses instead of brackets).
len	number of items in a variable	*a = [10, 20, 5]* *b = len(a)* Value of *b* is 3
slice	datatype consisting of start point, end point, and step (1 by default), used for example to crop images	*a = slice(1,5)* Make a slice containing integers from 1 to 4
append	add an item to the end of a list	*a = [1,2,3]* *a.append(4)* *a* is now [1, 2, 3, 4]
//	integer division	*a = 3//2* *a* is 1
ndim	number of dimensions in a NumPy array	*a = np.array([[[1,2],[3,4]],[[5,6],[7,8]]])* *b = a.ndim* *b* is 3
np.argwhere	find indices of NumPy array elements that are non-zero	*a = np.array([1,1,0,1,2])* *b = np.argwhere(a = = 0)* *b* is a NumPy array containing only one value: two
axis	define NumPy array axis in some operations; 0 = major/primary axis	*a = np.array([[1,1,0,1,2],[1,0,1,1,0]])* *b = np.*max*(a)* *c = np.*max*(a, axis=0)* *d = np.*max*(a, axis=1)* *b* is 2 *c* is np.array ([1,1,1,1,2]), i.e., column-wise maxima *d* is np.array ([2.1]), i.e., row-wise maxima
np.isin	check if a value appears in a NumPy array	*a = np.array([[1,1,0,1,2],[1,0,1,1,0]])* *b = np.isin(1, a)* *b* is True because 1 appears at least once in *a*
shape	shape (i.e., length of every dimension) of a NumPy array	*a = np.array([[[1,1,0,1],[1,0,1,1]],* *[[2,1,2,2], [1,2,1,1]],* *[[3,4,4,3], [5,0,0,2]]]) b = a.shape* *b* is (3, 2, 4)
np.array	NumPy array, an array datatype used by NumPy	*a = np.array([1,2])* Make a one-dimensional NumPy array consisting of two elements: numbers one and two
T	transpose a NumPy array	*a = np.array([[1,2],[3,4]])* *b = a.T* *b* is np.array ([[1,3], [2,4]])
enumerate	make (index, value)-pairs of a list	*a = [10,20]* *b = enumerate(a)* If *b* were converted to a list, it would be [(0, 10), (1, 20)],
np.ndindex	n-dimensional iterator containing indices of a NumPy array	*a = np.array([[5,25,30],[10,2,1]])* *b = list(np.ndindex(a.shape))* *b* is [(0, 0), (0, 1), (0, 2), (1, 0), (1, 1), (1, 2)]
product	cartesian product from the itertools Python package; the “repeat” argument can be used to define the number of repetitions for each element	*a = [1,2]* *b = [‘a’, ‘b’]* *c = product(a, b)* *d = product(a, b, repeat=2)* If *c* is converted to a list, it will be [(1, 'a'), (1, 'b'), (2, 'a'), (2, 'b')] If *d* is converted to a list, it will include 16 combinations from (1, ’a’, 1, ’a’) to (2, ’b’, 2, ’b’)
vectorize	vectorize a scalar function; this is used in label reduction (described in detail in Section 2.4)	*def add1(x):* *return x + 1* *vec_add1 = np.vectorize(add1)* *a = np.array([1, 2, 3, 4, 5])* *b = vec_add1(a)* *b* is np.array ([2, 3, 4, 5, 6])
lambda	anonymous, often short, function	cube = lambda x: x**3 *a = cube(2)* *a* is 8
index	location of an item in a list	*a = [6, 5, 4]* *b = a.index(5)* *b* is 1

The mathematical justification for all three decomposition methods lies in array decomposition, block identification, and efficient storage. To elaborate, by decomposing the input array into uniquely labeled contiguous blocks containing only foreground elements, the solutions allow for efficient array manipulation and analysis because the contents are broken down into smaller, more manageable blocks that are constructed around foreground elements and can be identified and represented by their position and dimensions. This provides for easy access and manipulation of image data. Put differently, by performing this decomposition, the methods facilitate efficient storage and retrieval of information, theoretically simplifying and speeding up data analysis.

### 2.2 Test procedure

The dataset the developed methods were made with and tested on consists of 13 synthetic n-dimensional binary images, where n ∈ {1, 2, 3, 4} (Fig. 2). In each test image, zero-value elements represent the background, and all the other elements belong to the foreground, thus being parts of objects. Although most use cases of rectangular partition use 2D and 3D data, a few examples of 1D and 4D arrays exist in the set to test and demonstrate the capabilities of the developed algorithms. The “Randomly Generated” shape in Fig. 2 was generated randomly, and all the other arrays were created manually. “Squares” and “Cubes” test the capability of the algorithms to handle data with multiple objects. All the arrays with “Holed” in their names are used to see if shape orientation affects partition results.

The tests were done on a Dell Precision 7520 laptop.

**Fig. 3:**
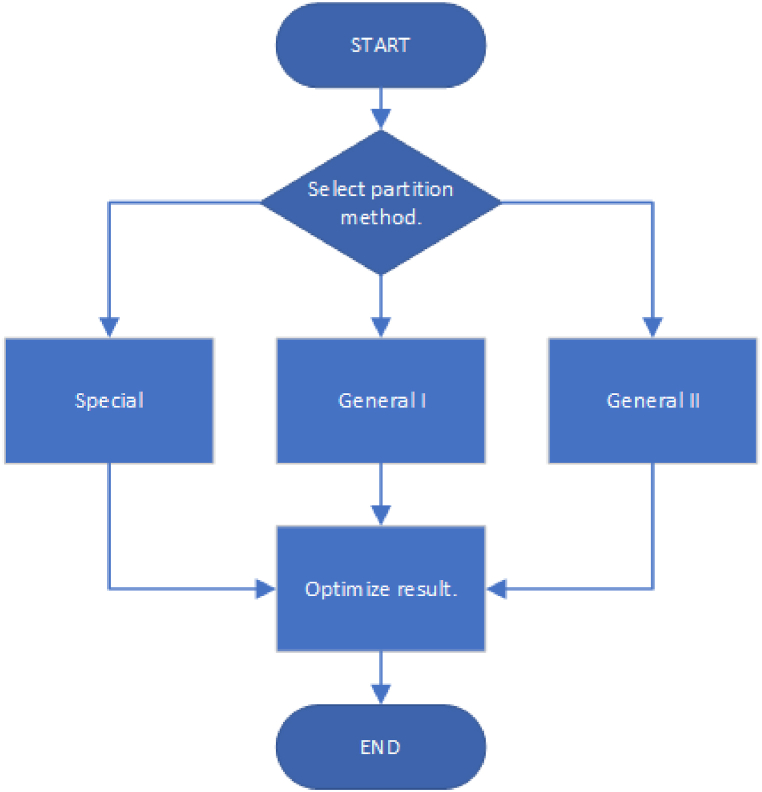
Test process. The same optimization procedure is used regardless of the selected partition method.

The test procedure (Fig. 3) is as follows: each array in the input data (Fig. 2) is given individually to each partition method. The starting point is picked randomly from all object elements for both General solutions; after a rectangular shape has been successfully extracted, a new point is chosen randomly until there are no more unvisited object elements. The results of both Special and General solutions are optimized so that extracted objects are merged whenever possible, and their assigned labels are recalculated so there are no skipped values. Each input array is partitioned five times using each method. This is to investigate the effect of randomness in object participation and to improve the testing process thoroughness. The execution times of testing and its phases are measured, and key performance indicators used for evaluating the methods and their effectiveness are calculated from the results (Section 3). The partitioning is considered successful if the extracted shape only consists of foreground elements of the same label, unique to this sub-object.

### 2.3 Solutions

#### 2.3.1 Special Solution

Arguably, the simplest way to implement rectangular partitioning for n-dimensional images containing only rectilinear shapes that may or may not have holes is to iterate through the image sequentially in one pass. The advantages of this method include ease of implementation, predictability of results, and repeatability of the process. The most obvious disadvantage is the lack of random access; the entire array must be traversed even when analyzing only a part of the image would be desirable.

The procedure for the Special solution for rectangular partition is presented in Algorithm 1. It takes one argument – the image (array) that will be processed – and returns an array containing rectangular subobjects with unique labels and a list of starting-point coordinates for each subobject. The output and input arrays are of equal shapes and sizes.

In short, the Special method works as follows: The initial step is discovering the first unvisited object element. After that, its position is used as the starting point for the new sub-object. The algorithm moves along every axis from this location until it hits the array boundary, a visited object element, or the background. The coordinates found this way are used as region edges and potential end-points of the sub-object. The last step is hole detection, which is realized simply by finding the lowest coordinates of background elements within the region and cropping such elements out of the sub-object if they are found; otherwise, the original region start point and end points are used. The process starts from discovering the first unvisited object element until there are no more such objects.

The formulae at the basis of Algorithm 1 focus on finding the coordinates of zero (background) and non-zero (foreground) elements, block slicing, and block size calculation. We can view the method presented in the algorithm as an application of set theory, linear algebra, and graph theory: The decomposition procedure constructs element sets from matrices (images) by using specific conditions to find coordinates. Linear algebra concepts, particularly matrix operations including element-wise comparison and slicing, are applied to represent and manipulate n-dimensional arrays (images). Additionally, the method utilizes graph traversal and iterative graph exploration to identify the connected components (extracted foreground subregions) within the image – the foreground elements serve as graph nodes.

Algorithm 1 “Special” solution for rectangular partition.

**Figure:**
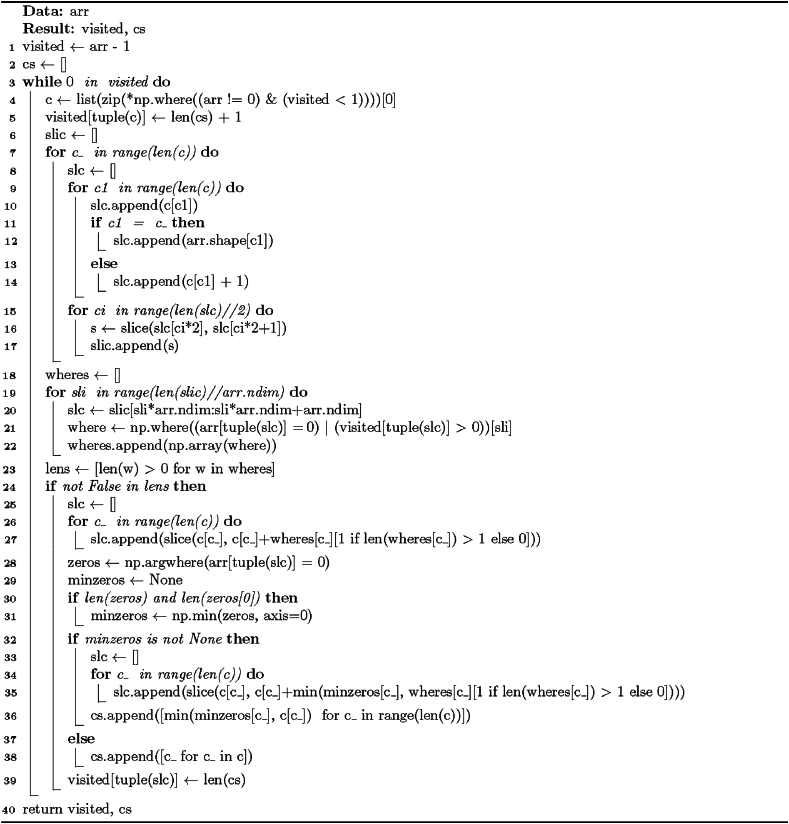

#### 2.3.2 General Solution I

This method can access array data randomly, meaning images need not be processed fully, and their shapes or objects can be partitioned in arbitrary order.

The sub-object extraction algorithm that forms the most crucial part of the solution is described as pseudocode in Algorithm 2. It is based on finding an enclosing (n-dimensional equivalent of) cuboid for the chosen object element and shrinking the enclosing cuboid until it no longer contains background elements. To simplify the process, the algorithm works on a copy of the array where it assigns an otherwise unused label to the target element (or “marks the target pixel with a special color”) and uses the “where” function of [16] to locate it every time the geometry of the enclosing cuboid changes. This minimizes the need for coordinate transforms.

Mathematically, Algorithm 2 involves an iterative refinement process and n-cuboid boundary adjustment to extract accurately a sub-shape from an image. The formulae used in the algorithm primarily focus on bounds adjustment such that the method obtains the region of interest with iterative boundary search (partly done in Algorithm 3) and a foreground cross-out operation (Algorithm 4). The shape and content of this extracted area contribute to determining the n-cuboid bounds while also involving setting new slices to refine said boundaries. The background of the algorithm lies in array manipulation, iterative optimization, set theory, and computational geometry. The procedure manipulates image indices and slices to extract and refine regions of interest and n-cuboid boundaries, leveraging concepts including subsets, minimum and maximum values, and Euclidean distances in locating background and foreground elements. The goal is to tightly enclose the extracted n-cuboidal sub-object encompassing the target element while excluding background elements, thus enhancing the accuracy and completeness of the partitioning.

The initial step is saving the location of the original target element, finding the enclosing cuboid (using Algorithm 3) in an uncropped array, cropping the image to the resulting coordinates, and storing the new shape to a variable.

After completing the above step, the algorithm checks if there are background elements in the cropped region. If this condition is met, the position information of the target element is updated to reflect the new coordinates. The cropped area is copied, and a cleanup operation (Algorithm 4) is performed on the copy to eliminate holes, concavities, angles, and intersections.

Next, the element located in the copy in the coordinates corresponding to the target position is checked. If the object in it was erased in the cleanup (i.e., is now zero), an element-wise subtraction is performed between the copy and the original cropped region with the former as the minuend and the latter as the subtrahend. The negative is taken from the result and used as the image array in subsequent steps, starting from finding new bounds (Algorithm 3) and cropping the region accordingly.

If the object in the target position in the copy was not erased, the copied array (post-cleanup) is used as the new image array in the remaining steps. New bounds are discovered (Algorithm 3), and the region is cropped to reflect these.

If the cleanup did not help crop the region (its shape did not change), another procedure is attempted: the cropped area is split into 2^n^ sub-regions, where n is the number of dimensions in the array. The position of the target element is used as the pivot and is included in every sub-region. For each split array, the minimum and maximum coordinates of background elements are saved to **MNS** and **MXS**, respectively, and used as a foundation of two new lists: one with minima of maxima and the other with maxima of minima (Equation (5) and Equation (6)):

(5) $$\begin{array}{c} \mathrm{mins}\left[n\right]=\mathrm{MXS}\left[2*n+1\right]\left[n\right]\;\text{for}\;n=1,2,\ldots ,m-1\text{,} \end{array}$$

and

(6) $$\begin{array}{c} \mathrm{maxs}\left[n\right]=\mathrm{MNS}\left[2*n\right]\left[n\right]\;\text{for}\;n=1,2,\ldots ,m-1\text{,} \end{array}$$

where m is the number of dimensions in the cropped region. These two lists now consist of per-axis minimum and maximum background element coordinates compiled from each sub-region and are combined into a single list of lists where minima are the first and maxima are the second elements. This list, whose length is equal to the number of dimensions in the cropped region, is iterated through, and target element coordinates and a constant value of one are added to the maxima. Three values are checked on each iteration: minimum, target element coordinate, and the adjusted maximum. The smallest of these is chosen as the start and the largest as the end for a slice of the current axis. A tuple is formed from the slices created when processing the list and used to clip the cropped region further.

If splitting the cropped region and using a combination of minimum and maximum background coordinates could not reduce the area, the position of the target element is compared against the largest coordinate in the cropped region (for example, the bottom right corner in 2D arrays). If the two are equal, the distances between this point and all the background elements are computed by summing the absolute values of the per-axis coordinate differences and taking the square roots of the results. The background element with the longest distance to the target point is used as a reference (the shortest distance would often result in too small areas): the region is further cropped by using its coordinates as the starting point, while the target element serves as the endpoint – but only if all dimensions of the resulting area are one or higher. This step is meant to crop the region so that edges that only consist of object elements ideally get cut out, and the resulting clipped array can be processed further in the next iteration.

The final recourse (when the region still has the same size as in the second step or the previous process would have caused it to have zero in its dimensions) is to pick the coordinates of the target element and nothing else, that is, a rectangular sub-object one unit long in every dimension.

The variable containing the shape stored in the first step is updated with that of the cropped region.

The steps above, starting from checking the presence of background elements in the region of interest, are repeated until this condition is not met.

Once the sub-object has been extracted, its coordinates are transformed back by subtracting the location of the new target (in the sub-shape) from the old position (in the original array) and constructing a tuple of slice objects with the coordinate differences as the starts and the sum of them and sub-object dimensions as the ends. This tuple now contains the global coordinates of the extraction result.

The code in Algorithm 2 is executed with randomly chosen foreground elements as the target pixels until the whole array is processed. Each time an enclosing cuboid is obtained, the region of the array corresponding to its coordinates is set to the background. This step reduces the possible foreground (or target) elements on every iteration.

The boundary-finding procedure (Algorithm 3) traverses the array in both directions along every dimension until it hits an array bound or a background element. This is like the procedure in the Special solution except bidirectional and does not consider visited elements.

The mathematical justification of Algorithm 3 comprises n-cuboid bound identification and iterative search along each dimension. The first is essential for isolating sub-objects within the image, and the second ensures the n-cuboid bounds the foreground elements in all directions. The formulae behind the algorithm include coordinate update and n-cuboid representation. In this context, the former adapts the minimum and maximum coordinates of the n-cuboid by foreground element positions, whereas the latter builds a structured representation of the n-cuboid. Algorithm 3 uses coordinate manipulation (processing array indices to access specific image elements and ensuring the accuracy of the n-cuboid bounds) and iterative search strategy (iterating over each dimension to search for foreground and background elements around the target element, guaranteeing the completeness and efficiency of the bounding box calculation). In summary, the algorithm employs concepts from linear algebra, iterative search strategies, and geometry.

**Fig. 4:**
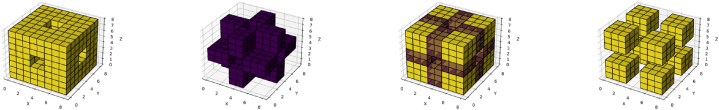
An example of the non-zero cutout process. From left to right: Input, cutout regions computed from background elements (said elements excluded from the picture for clarity), cutout regions superimposed on input, output with cutout regions set to background elements.

The cleanup operation (Algorithm 4) works as follows: the locations of background elements in the array are gathered, and for each such element, n regions of interest are formed, with n being the number of dimensions in the array. Each region spans across the entire array along one axis but is only one dimension long along the rest. The array parts corresponding to the formed areas are set to background elements. An example using the “Holed” image (with the surrounding background already cropped out) from the test dataset is given in Fig. 4.

Algorithm 4 has a mathematical background in set theory and matrix manipulation techniques, specifically in-place modification: the function efficiently uses array indices and slices to identify and modify background-associated elements. The manipulation operations ensure these elements do not affect subsequent partition steps, which is crucial for accurate decomposition and isolating relevant regions of interest (foreground sub-objects). A critical step in the process is slicing for cross-out, where the algorithm constructs slices to cover entire axes containing background elements, efficiently identifying and modifying foreground elements associated by axis with each background element. Although creating a copy of the image in Algorithm 2 for Algorithm 4 to work on requires additional memory, in-place manipulation still reduces mathematical complexity and enhances efficiency, especially for large images. In short, the justification for Algorithm 4 lies in its efficiency in modifying elements associated with the background so that even shapes with concave angles can be decomposed accurately.

Algorithm 2 “General” solution I for rectangular partition.

**Figure:**
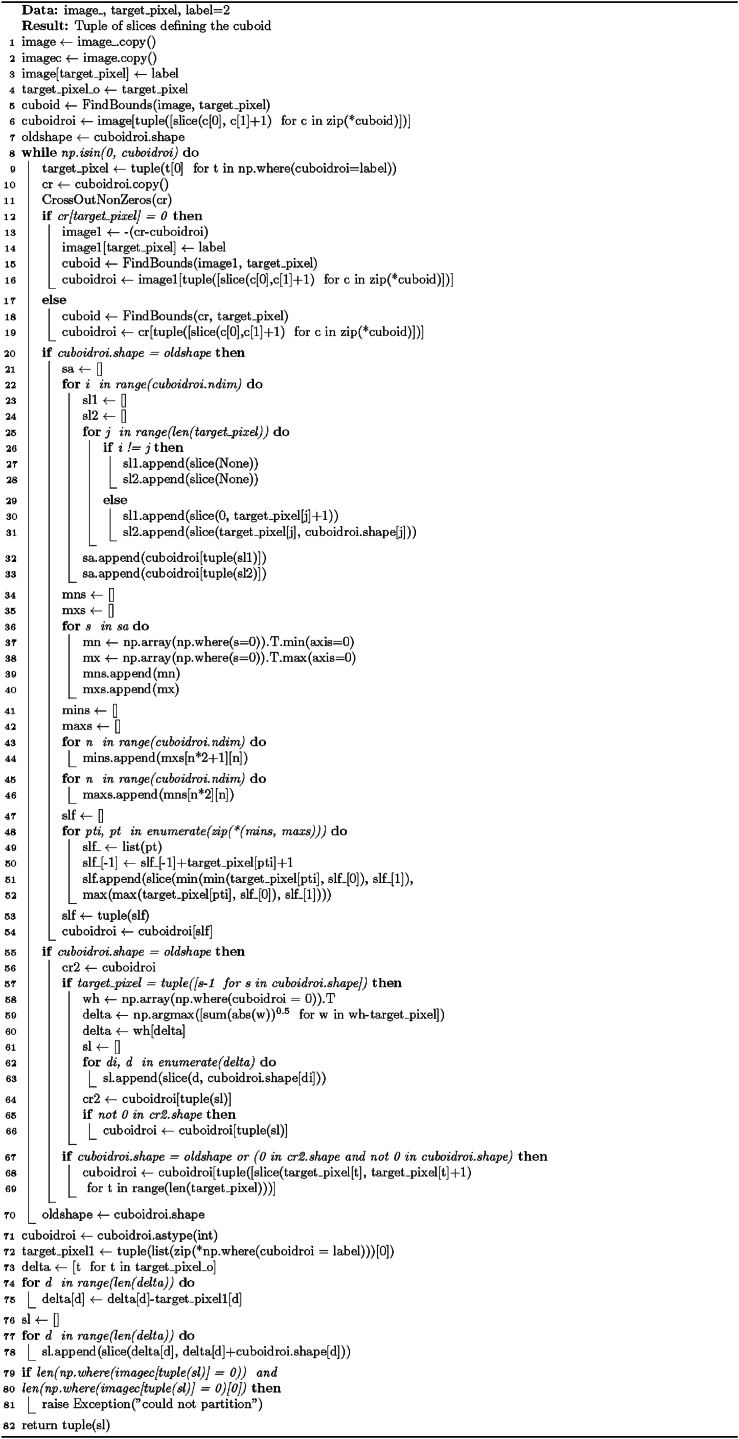

Algorithm 3 Auxiliary method FindBounds for boundary-finding.

**Figure:**
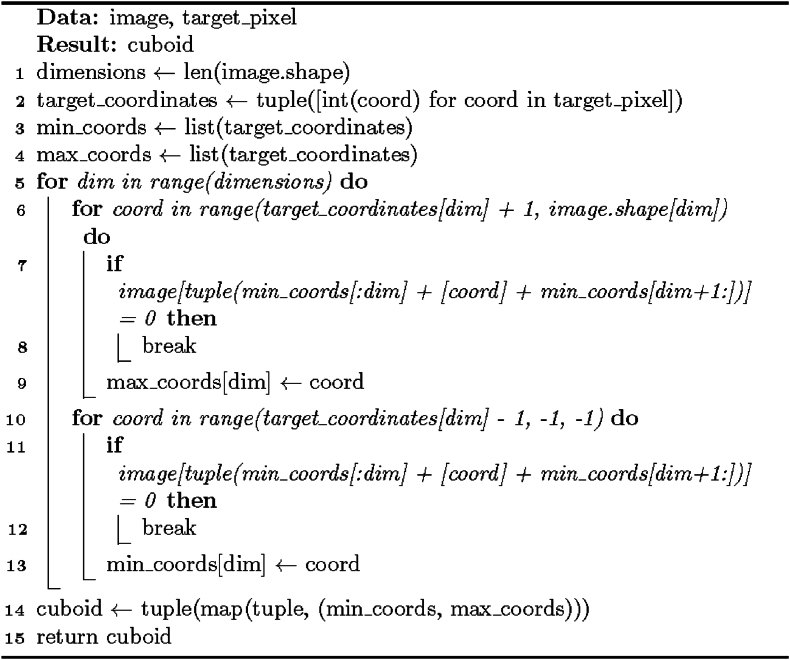

Algorithm 4 Auxiliary method CrossOutNonZeros for altering shape geometry in an array. The operation is done in place.

**Figure:**
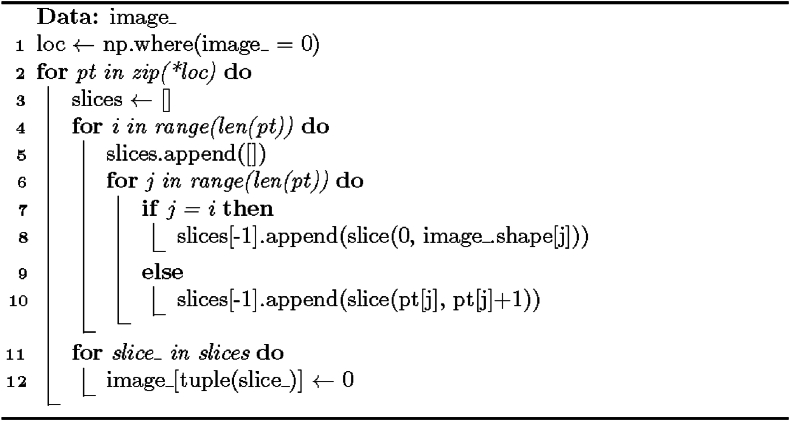

#### 2.3.3 General Solution II

Like General Solution I, this method can access data randomly. However, the most vital difference to the other solution is that this second procedure uses template-matching to find the sub-object boundaries. A template here is a rectangular shape with the same number of dimensions as the array. Each element in the region covered by the template must be part of an object, and the largest template area enclosing no background elements is chosen as the region of the extracted sub-shape.

The templates are formed by calculating all possible permutations of shapes up to the dimension set based on the maximum number of consecutive foreground elements found on each axis. For example, for a 2D array where this value is two on both axes, the largest template size would be 2*2 elements, and the outcome – excluding templates with zero in their shape – would be four permutations: (1, 1), (1, 2), (2, 1), and (2, 2). A pseudocode representation of this calculation is in Algorithm 6. The product function from the itertools software package that is part of the standard Python distribution is used to get all permutations.

The upper bound for the template sizes is calculated as follows: as a pre-processing step, the array is essentially sliced along every axis into pieces with the length of the axis as one dimension and zeros as others. For a 3D array having 3*3*3 elements, this would produce slices of 3*1*1, 1*3*1, and 1*1*3 units, and there would be 9 (3*3) slices of each type. However, the slices are formed as 1D arrays, so the example input would produce nine 3-unit-long slices along each axis, so 27 arrays in total. The slicing process is in pseudocode in Algorithm 9, and the code for calculating dimension and shape permutations for it is in Algorithm 7. Each slice returned by the process has its axis ID attached. Once the slices have been formed, the template boundary calculation can resume. A list **l** of the maximum number of consecutive foreground elements on every axis is created, and each value is initialized to zero. Next follows the processing of the slices. The axis ID is read, and the positions of elements of the background elements are discovered. If the slice only has foreground elements, its maximum number is set to its length. If there is one background element, the maximum number of the slice is set to one. Otherwise, the maximum of the absolute values of differences between the foreground element positions is used. This would be three for a slice **a** = [0,1,0,1,1,1,0]. The maximum value of the slice is compared to that saved to the index corresponding to the axis ID in the list **l** created before processing the slices. If this maximum exceeds the stored value, **l** is updated to hold the new value instead. After processing all the slices, the function returns a list containing the lengthiest sequence of foreground elements for each axis. A pseudocode representation of this procedure is in Algorithm 8.

**Fig. 5:**
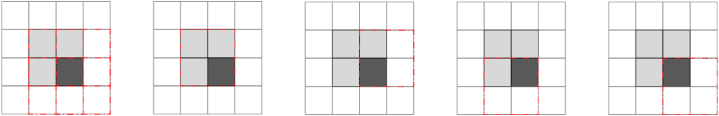
Example of permutations for a template size of 2x2 elements and on a 2D image with a target element. The leftmost array has all possible permutations stacked, while each possible permutation is presented individually in the others.

A simple example of permutations is provided in Fig. 5, where a 4*4-element 2D image with an object of 2*2 elements (gray squares) is surrounded by the background (white squares). The bottom-right part of the object has been chosen for the starting point; this is highlighted in darker gray than the rest of the shape. The dotted lines show the possible locations of the 2*2 template when the starting point is included in the region of interest. The leftmost part of Fig. 5 displays all possibilities at once; the result looks like a 3*3 grid. The other four parts show each template position separately. The second part from the left is the ideal solution because the area covered by the template only contains elements belonging to the object.

Once the maximum number of consecutive foreground elements on each axis has been computed, the output has been used to set the upper bounds for template creation, and the templates have been created, the sub-object extraction begins. For each template, possible starting positions are computed so that the target position is included, and secondly, there are no reads out of the array. The first criterion reduces processing time by avoiding unnecessary analysis of regions that may not be part of the currently targeted (sub-)object. The template is fitted for each remaining starting position: a sub-array is produced by using the coordinates of the location and template dimensions, and if all elements in this sub-array belong in the foreground, the sub-array size is compared to that of the best fit so far. If the size is larger, the best fit is updated to reflect this: it contains the new size, the coordinates and dimensions of the sub-array, and the template shape. Once the list of templates has been exhausted, the best fit is returned (Algorithm 5.).

The core of the partition process is described in Algorithm 5. Like the principal part of General Solution I, the code in Algorithm 5 is executed with randomly chosen foreground elements as the target pixels until the whole array is processed. Each time a sub-shape has been extracted, the region of the array corresponding to its coordinates is set to the background. This reduces the possible foreground (or target) elements on every iteration.

From a mathematical perspective, the rationale behind Algorithm 5 lies in contiguous block identification to detect the most sizable such region (a foreground object or sub-object). The method employs a systematic search to achieve this goal by iterating over possible dimensions and starting indices, using formulae that manage block size computation (based on array analysis, matrix manipulations, and set theory) to check if the block contains only foreground elements. The iteration techniques ensure computational efficiency, especially for large images: using permutations (Algorithm 6, Algorithm 7) and the lengthiest per-axis non-zero (foreground) element sequences (Algorithm 8) as references when computing the n-cuboid sizes and positions and using the fast n-dimensional index operator implemented in NumPy. Additionally, Algorithm 5 uses Algorithm 9 and mathematical concepts related to it in some of its steps.

The mathematical background of Algorithm 6, Algorithm 7 is primarily in combinatorial analysis and related techniques and secondarily in matrix operations (namely, obtaining dimensionality and creating slices for the permutations). Using cartesian products of the inputs, the objective of the algorithms is to systematically generate comprehensive and unique permutations that explore different array shapes, structures, and element subsets along various dimensions. The methods ensure all possible combinations, either of array dimensions within specified limits (Algorithm 6) or slices along dimensions (Algorithm 7), are explored. The algorithm outputs later find application in array (image) subset analysis and manipulation.

Algorithm 8 relies on algorithmic techniques, numerical analysis, set theory, and linear algebra (matrix operations) to identify sequences of consecutive non-zero (foreground) elements along each axis of the array (image) and to compute the maximum length of the resulting lines through axis-wise systematic analysis and mathematical techniques. These methods make image slice computations used in the sequence identification task efficient and accurate. In addition to matrix operations and set theory, finding runs of foreground elements and computing differences between their indices are utilized. The result provides information about the spatial distribution of the foreground elements within the image.

Algorithm 9 is based mathematically on combinatorial analysis, which generates all possible dimension options and slice permutations, and an iterative approach to explore possible choices and rapidly generate comprehensive combinations for slicing along each dimension. Enumerating these slice permutations ensures a thorough slicing combination coverage, making the exploration of array element subsets along different dimensions possible. The objective is to facilitate the array subset analysis and manipulation necessary for polygon partitioning in General Solution II.

Algorithm 5 “General” solution II for rectangular partition.

**Figure:**
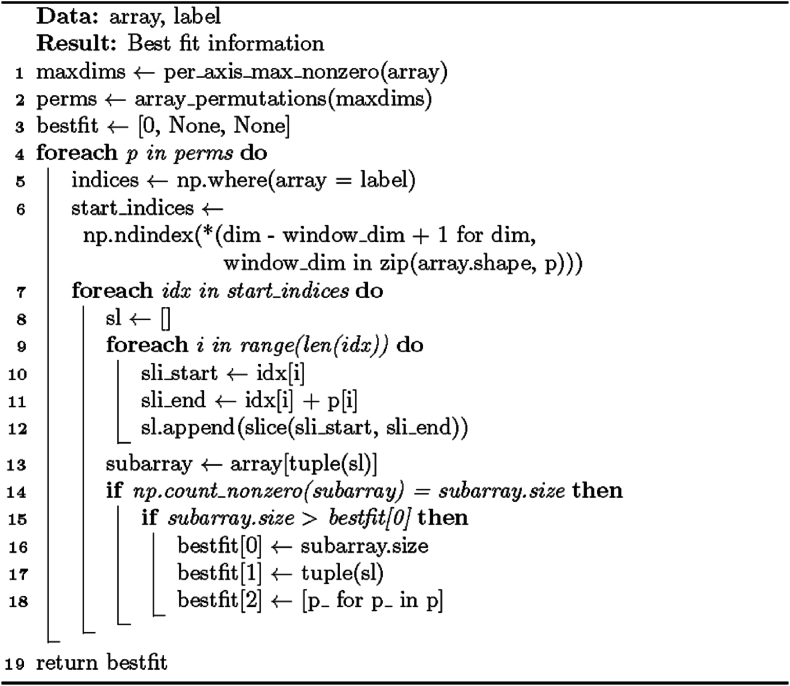

Algorithm 6 Auxiliary method "array_permutations".

**Figure:**
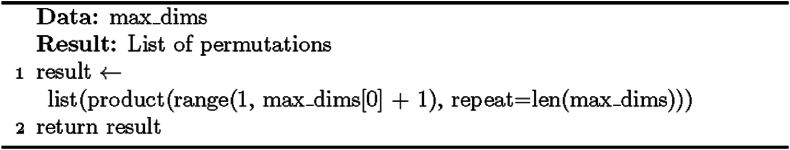

Algorithm 7 Auxiliary method "create_permutations".

**Figure:**
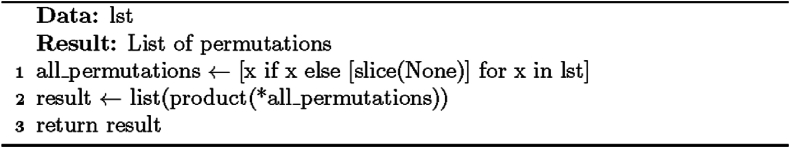

Algorithm 8 Auxiliary method "per_axis_max_nonzero".

**Figure:**
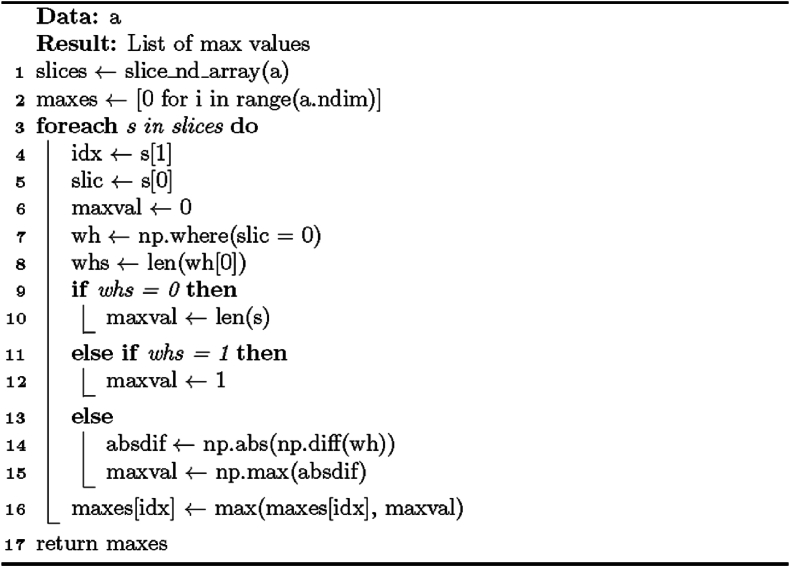

Algorithm 9 Auxiliary method "slice_nd_array".

**Figure:**
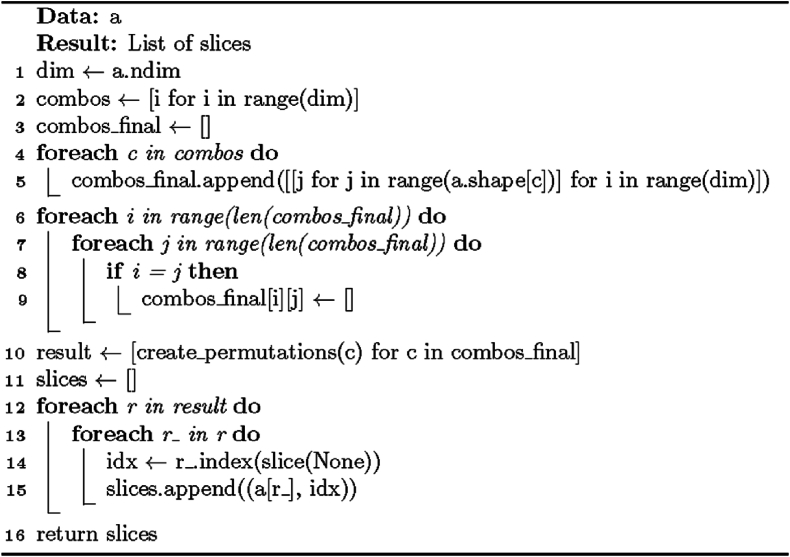

### 2.4 Optimization

**Fig. 6:**
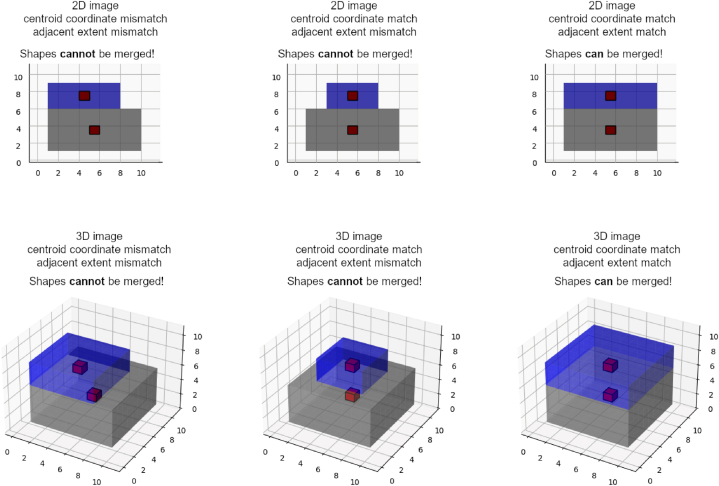
Conditions for merging adjacent shapes.

Partition results can be optimized by merging adjacent rectangular shapes. This, however, can only be done if two conditions are met: Firstly, the shapes must share their centroid coordinates in all dimensions except one, and secondly, their boundaries corresponding to shared coordinates must have equal length. The merging process is repeated until no more shapes that meet the conditions are found. A non-exhaustive list of examples of shapes that can and cannot be merged is shown in Fig. 6: three pairs of 2D shapes and three pairs of 3D shapes.

Results can be further cleaned after optimization by reassigning unused labels of extracted shapes. For example, if the optimized array only has labels 0 (background), 1, 3, and 4, these can be reassigned to 0, 1, 2, and 3.

The optimization process is presented in pseudocode in Algorithm 10, and the evaluation of the partition, optimization, and label reduction procedures is described similarly in Algorithm 11.

Mathematically, the justification of Algorithm 10 is in processes that guarantee precise label comparison and reduction. It involves comparing the centroids of labeled sub-objects within their bounding boxes and the dimensions of these boxes to confirm a distinct separation that allows for merging only when regions are adjacent and proportionate in related dimensions. Additionally, it can minimize label values without compromising the integrity of the optimization. The foundation of Algorithm 10 exists in calculating and comparing centroids, determining bounding box sizes, and optionally reducing labels. Its mathematical framework includes analyzing centroids and bounding box dimensions to evaluate the merging suitability of labeled subregions. The analysis ensures spatial coherence and remaps labels to maintain segmentation accuracy while preserving the spatial relationships between regions. This dual approach of centroid analysis and label remapping ensures both the accuracy of segmentation and the efficiency of label use.

A critical aspect of Algorithm 10 is its dependency on an evaluation function (Algorithm 11) to check that the optimization process has not introduced errors or inaccuracies. The mathematical justification and background of the evaluation (Algorithm 11) consist of region contiguity and validity realized through iterative region analysis for studying each labeled region and ensuring comprehensive coverage of all areas, the combination of array operations and set theory for efficient region extraction and manipulation while also ensuring computational efficiency, and Boolean logic to determine the success status of the decomposition evaluation, indicating clearly whether the outcome is valid. Therefore, the function verifies that the regions have neither gaps nor disjoint components and ensures that each labeled region contains only one unique non-zero label value, indicating that all regions solely consist of foreground elements and that the optimized image contains no mixed nor overlapping sub-objects.

Algorithm 10 Optimization procedure of partitioned shapes.

**Figure:**
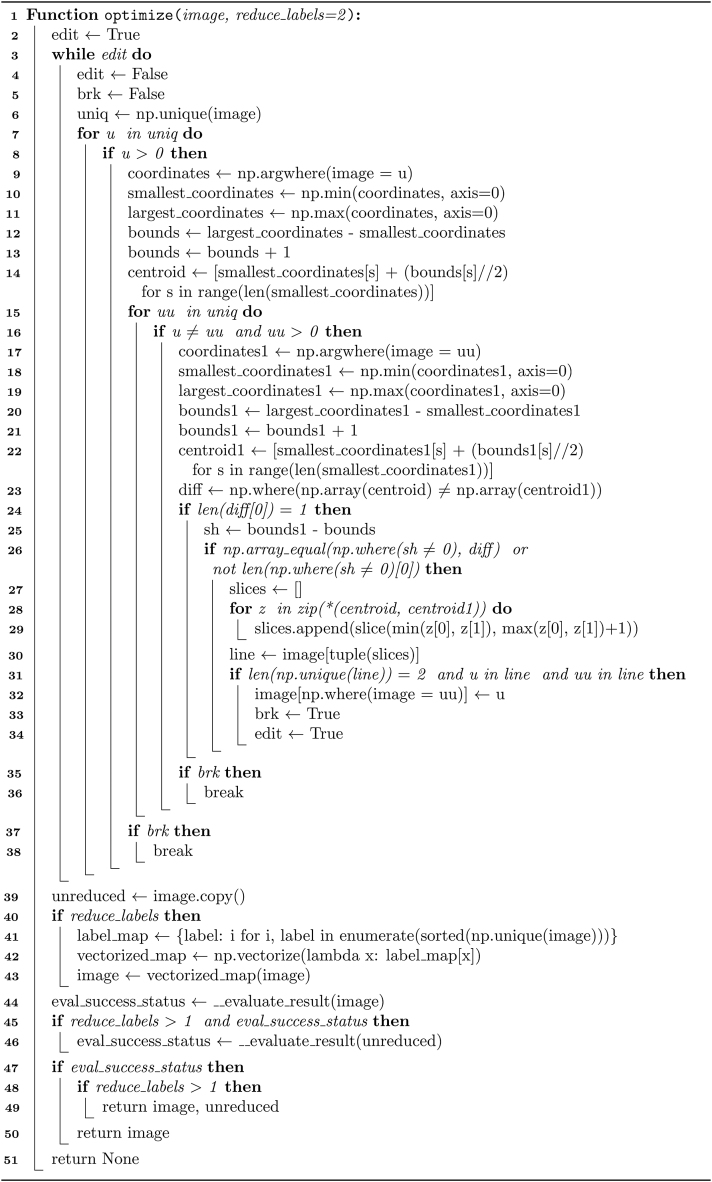

Algorithm 11 Evaluation of partition and optimization results.

**Figure:**
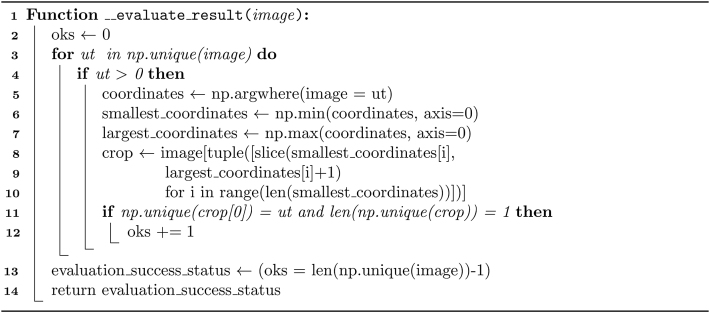

## 3 Experimental results

All three solutions processed the test dataset successfully, with neither overlapping shapes nor background elements in any of the extracted rectangular shapes.

Six metrics were chosen to evaluate the methods, and three operations were applied to each, resulting in 18 key performance indicators. The operations – minimum, maximum, and average – were performed for each array and method by finding the smallest and largest values and computing the arithmetic mean from the corresponding metrics (every array is processed five times with all methods). For example, the first minimum time for General II is 0.321 s. Out of the five test runs, this is the shortest processing time of the method for the first array (named “Box 9 × 9 × 9”). In addition to execution time, the number of labels in the results, region size, and reduction count are used. The first two of these are calculated before and after optimization. Region size is determined solely by the number of elements in the extracted shape. Reduction count is the set difference between the labels in optimized and unoptimized results.

In addition to the 18 per-array and per-method metrics above, some indicators for the entire dataset were computed and used in the evaluation (Equations (7), (8), (9)). These are per-method averages for times, optimizations for shape sizes and labels, and reductions for every method. The first of these is simply the arithmetic mean of execution times. Average per-method optimization for shape sizes $o_{s}$ is calculated by using

(7) $$\begin{array}{c} o_{s}=100*\bar{s}-100\text{,} \end{array}$$

where $\bar{s}$ denotes the arithmetic mean of size ratios for the respective method (Special, General I, or General II). Conversely, average per-method optimization for label counts $o_{l}$ is calculated with

(8) $$\begin{array}{c} o_{l}=100-100*\bar{l}\text{,} \end{array}$$

where $\bar{l}$ is the arithmetic mean of label ratios for the Special, General I, or General II method. Finally, reduction r is simply

(9) $$\begin{array}{c} r=100*\bar{r}\text{,} \end{array}$$

with $\bar{r}$ denoting average reduction ratios. Size ratios are quotients of sizes of extracted shapes (expressed in the numbers of elements belonging to each shape) in optimized and unoptimized arrays. Label ratios are calculated similarly. A reduction ratio is obtained by dividing the reduction count by the region size of the optimized array.

The computed key performance indicators for each array in the dataset are gathered in Table 2. All values are rounded to three decimals. If the fourth decimal is five or more, the value is rounded up; otherwise, rounding down is used. The indicators for all arrays are listed in the following order using row-major layout: “Box 9 × 9 × 9,” “Holed,” “Holed with Planes YZ,” “Holed with Planes XZ,” “Holed with Planes XY,” “Cross 3D,” “Cross 2D,” “Randomly Generated,” “Steps,” “Squares,” “Cubes,” “Hypershape,” and “Lines.” Indicators for the whole dataset and the number of successful tests for each partition method are listed in Table 3. The total number of tests for each method is 65, the product of the input array count and the number of tests per array (13 and 5, respectively).

**Table 2:** Test results for each array in the dataset.

	Special	General I	General II
Minimum time (s)	0.001, 0.021, 0.017, 0.016, 0.007, 0.003, 0.002, 0.029, 0.005, 0.001, 0.001, 0.008, <0.001	0.001, 0.016, 0.005, 0.010, 0.011, 0.003, 0.002, 0.005, 0.004, 0.001, 0.001, 0.008, 0.001	0.321, 3.636, 2.202, 1.691, 1.710, 0.699, 0.013, 0.027, 0.204, 0.006, 0.123, 10.832, 0.001
Maximum time (s)	0.009, 0.029, 0.023, 0.018, 0.008, 0.004, 0.002, 0.037, 0.007, 0.001, 0.001, 0.009, 0.001	0.005, 0.023, 0.024, 0.018, 0.024, 0.005, 0.003, 0.014, 0.007, 0.001, 0.002, 0.009, 0.001	0.359, 3.756, 2.270, 1.825, 1.808, 0.722, 0.015, 0.038, 0.240, 0.007, 0.131, 11.466, 0.002
Average time (s)	0.003, 0.025, 0.020, 0.017, 0.008, 0.003, 0.002, 0.034, 0.006, 0.001, 0.001, 0.008, 0.001	0.002, 0.020, 0.010, 0.014, 0.017, 0.004, 0.002, 0.010, 0.005, 0.001, 0.001, 0.008, 0.001	0.332, 3.692, 2.239, 1.763, 1.762, 0.712, 0.014, 0.032, 0.219, 0.006, 0.127, 11.026, 0.001
Min. labels before opt.	1, 21, 18, 18, 11, 5, 3, 22, 9, 2, 2, 7, 2	1, 12, 6, 8, 10, 5, 3, 8, 7, 2, 2, 7, 2	1, 15, 10, 6, 6, 5, 3, 10, 9, 2, 2, 7, 2
Max. labels before opt.	1, 21, 18, 18, 11, 5, 3, 22, 9, 2, 2, 7, 2	1, 16, 16, 13, 13, 5, 3, 13, 10, 2, 2, 7, 2	1, 15, 10, 6, 6, 5, 3, 10, 9, 2, 2, 7, 2
Avg. labels before opt.	1, 21, 18, 18, 11, 5, 3, 22, 9, 2, 2, 7, 2	1, 14.2, 9.2, 10.4, 11.8, 5, 3, 9.8, 8.2, 2, 2, 7, 2	1, 15, 10, 6, 6, 5, 3, 10, 9, 2, 2, 7, 2
Min. labels after opt.	1, 12, 9, 8, 7, 27, 3, 7, 9, 2, 2, 7, 2	1, 12, 6, 6, 6, 5, 3, 6, 7, 2, 2, 7, 2	1, 15, 9, 6, 6, 5, 3, 9, 6, 2, 2, 7, 2
Max. labels after opt.	1, 12, 9, 8, 7, 27, 3, 7, 9, 2, 2, 7, 2	1, 12, 10, 6, 10, 5, 3, 10, 10, 2, 2, 7, 2	1, 15, 9, 6, 6, 5, 3, 9, 6, 2, 2, 7,
Avg. labels after opt.	1, 12, 9, 8, 7, 27, 3, 7, 9, 2, 2, 7, 2	1, 12, 7.8, 7.4, 8.8, 5, 3, 7.4, 8.2, 2, 2, 7, 2	1, 15, 9, 6, 6, 5, 3, 9, 6, 2, 2, 7, 2
Min. region size before opt.	343, 20.571, 25.333, 25.333, 41.455, 27, 11, 1, 6.111, 9, 27, 20.571, 2.5	343, 27, 28.5, 35.077, 35.077, 27, 11, 1.692, 5.5, 9, 27, 20.571, 2.5	343, 28, 45.6, 76, 76, 27, 11, 2.2, 6.111, 9, 27, 20.571, 2.5
Max. region size before opt.	343, 20.571, 25.333, 25.333, 41.455, 27, 11, 1, 6.111, 9, 27, 20.571, 2.5	343, 36, 76, 57, 45.6, 27, 11, 2.75, 7.857, 9, 27, 20.571, 2.5	343, 28, 45.6, 76, 76, 27, 11, 2.2, 6.111, 9, 27, 20.571, 2.5
Avg. region size before opt.	343, 20.571, 25.333, 25.333, 41.455, 27, 11, 1, 6.111, 9, 27, 20.571, 2.5	343, 30.703, 55.462, 45.269, 39.042, 27, 11, 2.319, 6.840, 9, 27, 20.571, 2.5	343, 28, 45.6, 76, 76, 27, 11, 2.2, 6.111, 9, 27, 20.571, 2.5
Min. region size after opt.	343, 36, 50.667, 57, 65.143, 27, 11, 3.143 6.111, 9, 27, 20.571, 2.5	343, 36, 45.6, 50.667, 45.6, 27, 11, 2.2, 5.5, 9, 27, 20.571, 2.5	343, 28, 50.667, 76, 76, 27, 11, 2.444, 9.167, 9, 27, 20.571, 2.5
Max. region size after opt.	343, 36, 50.667, 57, 65.143, 27, 11, 3.143, 6.111, 9, 27, 20.571, 2.5	343, 36, 76, 76, 76, 27, 11, 3.667, 7.857, 9, 27, 20.571, 2.5	343, 28, 50.667, 76, 76, 27, 11, 2.444, 9.167, 9, 27, 20.571, 2.5
Avg. region size after opt.	343, 36, 50.667, 57, 65.143, 27, 11, 3.143, 6.111, 9, 27, 20.571, 2.5	343, 36, 60.149, 63.333, 53.707, 27, 11, 3.059, 6.840, 9, 27, 20.571, 2.5	343, 28, 50.667, 76, 76, 27, 11, 2.444, 9.167, 9, 27, 20.571, 2.5
Min. reductions	0, 8, 7, 7, 3, 0, 0, 4, 0, 0, 0, 0, 0	0, 0, 0, 2, 1, 0, 0, 1, 0, 0, 0, 0, 0	0, 0, 1, 0, 0, 0, 0, 1, 1, 0, 0, 0, 0
Max. reductions	0, 8, 7, 7, 3, 0, 0, 4, 0, 0, 0, 0, 0	0, 4, 4, 3, 4, 0, 0, 3, 0, 0, 0, 0, 0	0, 0, 1, 0, 0, 0, 0, 1, 1, 0, 0, 0, 0
Avg. reductions	0, 8, 7, 7, 3, 0, 0, 4, 0, 0, 0, 0, 0	0, 2.2, 1, 2.2, 2.4, 0, 0, 2, 0, 0, 0, 0, 0	0, 0, 1, 0, 0, 0, 0, 1, 1, 0, 0, 0, 0

**Table 3:** Average values and the number of successful tests for each partition method.

	Special	General I	General II
Average times	0.010s	0.007s	1.686s
Average optimizations (sizes)	43.96 %	11.07 %	5.56 %
Average optimizations (labels)	19.46 %	7.86 %	4.10 %
Average reductions	13.86 %	6.63 %	4.14 %
Successful tests	65/65 (100 %)	65/65 (100 %)	65/65 (100 %)

**Fig. 7:**
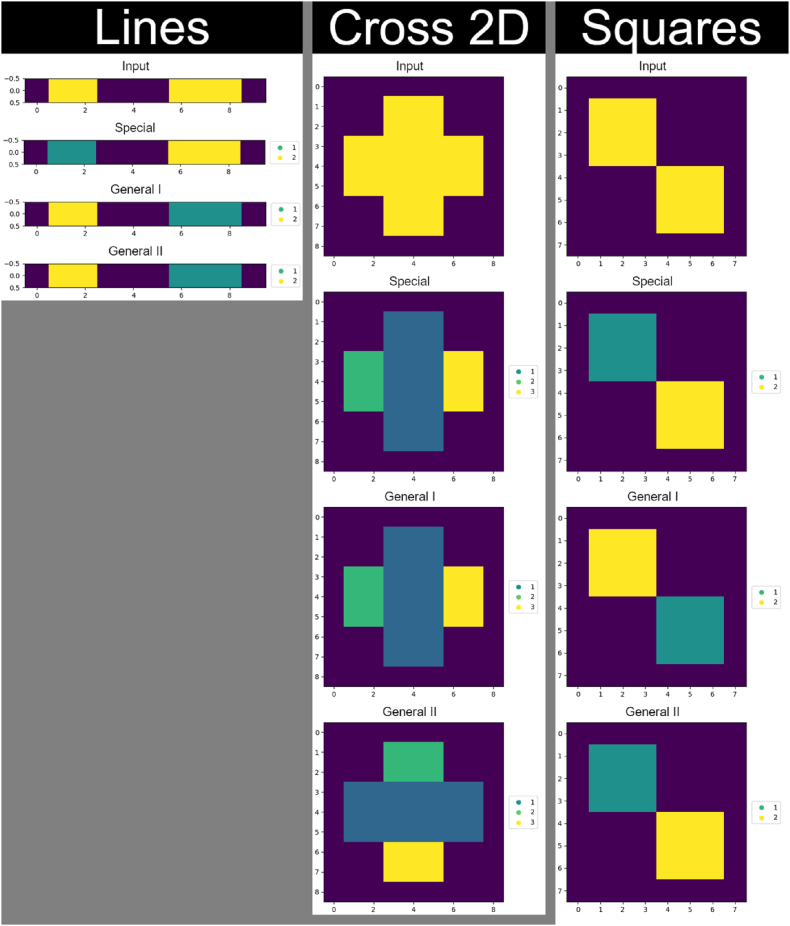
Results for the 1D (leftmost column) and 2D (central and rightmost column) shapes in the dataset.

**Fig. 8:**
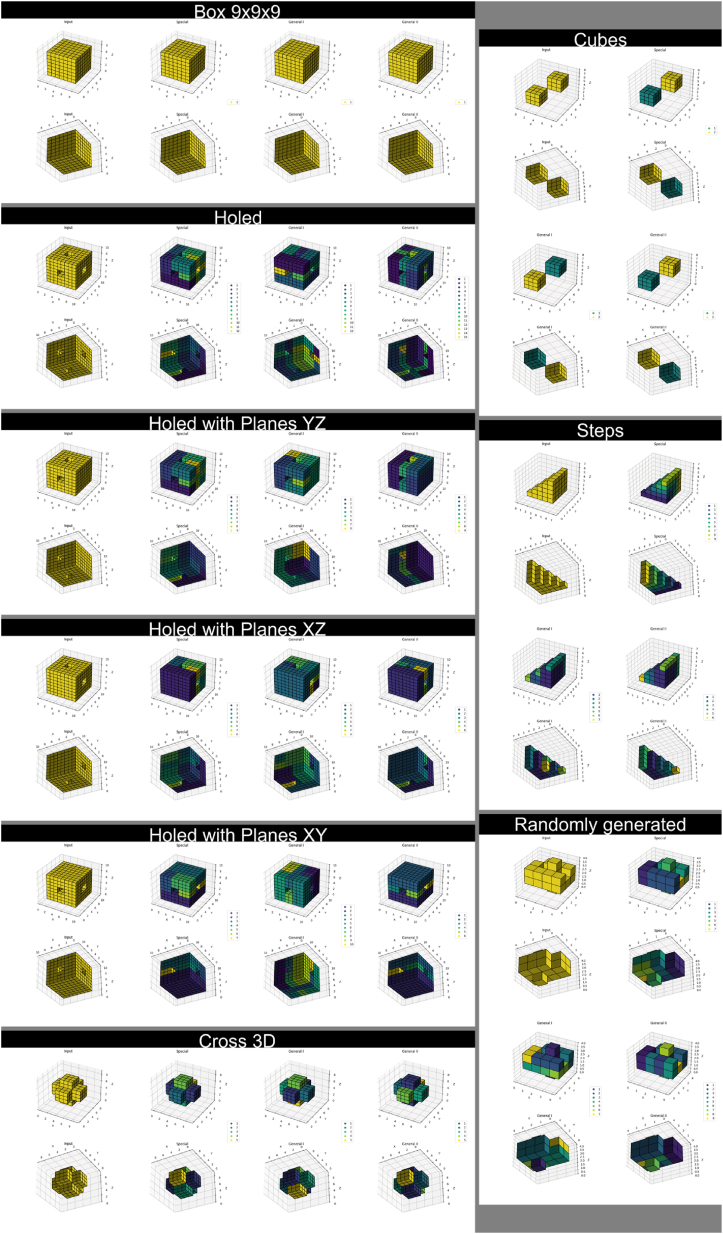
Results for the 3D shapes in the dataset.

**Fig. 9:**
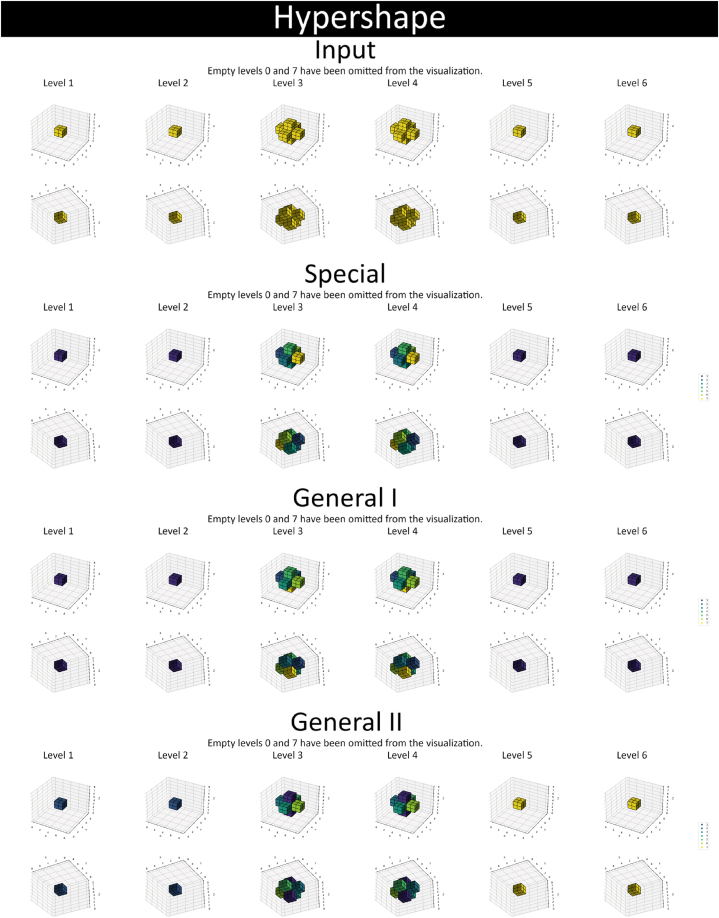
Results for the 4D shape in the dataset.

Example results of rectangular partition appear in Fig. 7, Fig. 8, Fig. 9, and Supplementary Fig. S1 provides an alternative illustration. These four figures demonstrate the outcome of the same test: the first in a series of five. Optimization and reduction were applied to the results before plotting. It should be noted some coordinate axes are swapped between Fig. 8, Fig. 9 and Supplementary Fig. S1 for 3D and 4D arrays; these swaps are listed in Table 4. In Fig. 9, the w-index is denoted by “Level” above the corresponding 3D slice. In Supplementary Fig. S1, the z-coordinate and w-coordinate are the vertical and horizontal indices of the 2D slice, respectively. Moving down or right increases their values. This applies to both 3D and 4D arrays.

**Table 4:** Axes in visualizations of results.

Fig. 8 (3D)	x	y	z	–
Supplementary Fig. S1 (3D)	z	y	x	–
Fig. 9 (4D)	x	y	z	w
Supplementary Fig. S1 (4D)	w	y	x	z

The sequential nature of the Special method is evident in Fig. 7, Fig. 8, Fig. 9, and Supplementary Fig. S1; the labels of extracted shapes grow along the coordinate axes. Conversely, partitions obtained through the General techniques have a more scattered label placement (Fig. 7, Fig. 8, Fig. 9). Comparing (for instance) the minimum, maximum, and average values of the metrics in Table 2 reveals that the General I method exhibits the most variability among the numbers. This observation may suggest that the procedure in question is the least predictable of the three ways to partition rectilinear shapes. The most significant connection discovered between shape orientation and partition results is the tremendously reduced (50–65 % on average) processing time of, and use of fewer labels (resulting in larger sub-objects) in, partitioning the shape named “Holed with Planes XY” when using the Special method.

**Fig. 10:**
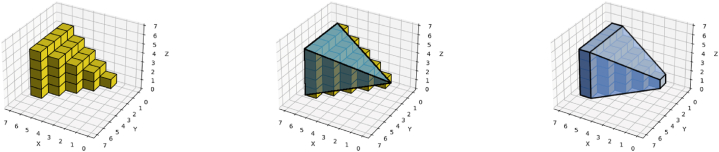
The original ”Steps” shape (left) and two manual ”devoxelizations” (center and right).

While the proposed algorithms are designed specifically for partitioning shapes consisting only of orthogonal, or 90-degree, angles and straight lines, it is possible to convert other kinds of shapes into rectangular and rectilinear forms, for example, by rotation for simple objects or using pixelation or voxelization for any object regardless of their complexity. The latter can be achieved with some conversion methods and tools software, for example [19] for 2D and 3D images and [20,21] for three-dimensional data. While this study employed no such methodologies or programs, the “Steps” shape in the dataset used for the tests can be considered analogous to a voxelization result of a 3D mesh originally consisting of one rectangular and rectilinear plus several oblique surfaces (Fig. 10). The decomposition results (Table 3, Fig. 8, Supplementary Fig. S1) suggest that the algorithms are likely to succeed in partitioning complex objects of this kind.

## 4 Conclusions

Rectangular partition is a fundamental geometry problem with many applications. To the authors’ knowledge, the solutions presented here are the first that work on n-dimensional rectilinear shapes, including those with holes. The proposed methods are possibly the earliest capable of processing shapes with four or more dimensions.

Each presented method has its advantages and disadvantages: the Special solution yields the most predictable results, and it is the easiest to implement, but it often loses in speed to the first General solution, and its use requires iterating through the whole input data. The first General solution performs fastest on experimental data, but its results are the least predictable. The second General solution is a tradeoff between speed, accuracy, and predictability of results. Despite its shortcomings, particularly in execution time, this method is still expected to perform reasonably effectively in the future due to advances in computational power. Some other criteria for General solutions in place of random selection might prove more effective solutions with more predictable results for partitioning shapes into rectangular subcomponents. One potential alternative is basing the choice of the next unvisited "target" element on the distance to the last extracted sub-object and values of neighbors of possible "target" elements. These criteria could be a basis for solutions that enable more optimal or application-appropriate sub-object extraction. Furthermore, the General methods might be sped up with parallelization or precalculated look-up tables.

## Funding

This research did not receive any specific grant from funding agencies in the public, commercial, or not-for-profit sectors.

## Ethics declaration

Informed consent was not required for this study because the data for this article do not include personal information. The study involved neither participants, patients, nor animals.

## Data availability statement

The data are encapsulated within the article. An alternative representation of the experimental results is provided in Supplementary Fig. S1.

## CRediT authorship contribution statement

**Ville Pitkäkangas:** Writing – review & editing, Writing – original draft, Visualization, Validation, Supervision, Software, Resources, Project administration, Methodology, Investigation, Formal analysis, Data curation, Conceptualization.

## Declaration of competing interest

The authors declare that they have no known competing financial interests or personal relationships that could have appeared to influence the work reported in this paper.

## Figures and Tables

**figs1:**
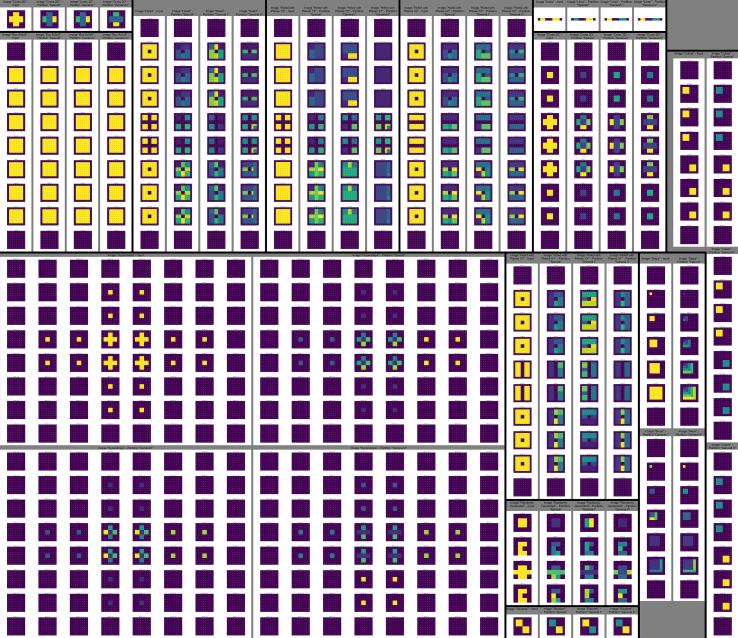
Results for the dataset as visualized in 2D slices.
